# Pregnancy and childbirth outcomes in women with myeloproliferative neoplasms—a nationwide population-based study of 342 pregnancies in Sweden

**DOI:** 10.1038/s41375-022-01688-w

**Published:** 2022-09-07

**Authors:** Anna Ravn Landtblom, Therese M.-L. Andersson, Anna L. V. Johansson, Sophia Brismar Wendel, Frida E. Lundberg, Jan Samuelsson, Magnus Björkholm, Malin Hultcrantz

**Affiliations:** 1 Department of Medicine, Solna, Karolinska Institutet, Stockholm, Sweden; 2 Department of Hematology, Karolinska University Hospital, Stockholm, Sweden; 3 Department of Medical Epidemiology and Biostatistics, Karolinska Institutet, Stockholm, Sweden; 4 Cancer Registry of Norway, Oslo, Norway; 5 Department of Clinical Sciences, Danderyd Hospital, Karolinska Institutet, Stockholm, Sweden; 6 Department of Hematology, Linköping University Hospital, Linköping, Sweden; 7 Department of Medicine, Myeloma Service, Memorial Sloan-Kettering Cancer Center, New York, NY USA

**Keywords:** Myeloproliferative disease, Epidemiology

## Abstract

Pregnancy and childbirth in women with myeloproliferative neoplasms (MPN) are reported to be associated with maternal thrombosis, hemorrhage, and placental dysfunction. To assess the risks of adverse events in pregnancy in women with MPN, we performed a large population-based study using Swedish health care registers, and included all pregnancies that had reached gestational week 22 (prior to 2008, week 28) during the years 1973–2017 in women with MPN. Control pregnancies were matched 1:1 for age, calendar year, and parity. We identified 342 pregnancies in 229 women with MPN. Preterm birth was significantly increased in pregnancies in MPN, 14% compared to 4% of pregnancies in controls (*p* < 0.001). Correspondingly, low birth weight (<2500 g) was also significantly increased in MPN pregnancies (*p* = 0.042). Stillbirth was rare, with two events (0.6%) in MPN, none in controls. Maternal thrombotic complications occurred in three (1%) of the pregnancies in MPN patients, compared to none in controls. Pregnancy-related bleeding affected 14% of pregnancies in MPN and 9% in controls (*p* < 0.110). Cesarean section was significantly more common in pregnancies in MPN. Incidence was 12.2 per 100.000 pregnancies. In summary, preterm birth was an important complication in MPN pregnancies, while maternal complications were less common than previously reported.

## Introduction

Myeloproliferative neoplasms (MPN) are a family of chronic hematologic malignancies, characterized by excess proliferation in myeloid cell lineages and/or excess fibrosis of the bone marrow [1]. MPNs consist of polycythemia vera (PV), essential thrombocytosis (ET), primary myelofibrosis (PMF), and MPN unclassifiable (MPN-U) [1]. Although MPNs are most commonly diagnosed in middle-aged and elderly individuals, 10–20% of patients with a newly diagnosed MPN are of fertile age [2–4]. The live birth rate in pregnant women with MPN is reported to be 65% in PV and 70% in ET, while pregnancy in women with PMF and MPN-U has mainly been described in case reports or smaller case series [3–6]. Complications described range from early miscarriages, repeated miscarriages, stillbirth, placental infarction, thrombosis, bleeding, preeclampsia, and preterm birth [3, 7–9]. Alimam et al. have in a British prospective and population-based study reported better outcomes, with a higher live birth rate (97%) in 58 pregnancies in MPN. In this study, the incidence of pregnancies in women with MPN per 100,000 pregnancies was 3.2 [7]. The rarity of pregnancy in patients with MPN has to a large extent limited systematical studies of outcomes and optimal management.

The purpose of this study was to assess pregnancy and childbirth outcomes in MPN patients in Sweden between 1973 and 2017 in a population-based setting, using Swedish healthcare registers and matched controls for comparison. We aimed to describe pregnancy outcomes, including obstetric and perinatal outcomes, quantify risks of adverse outcomes, and to estimate the incidence of pregnancy in women with MPN.

## Patients and methods

### Source population

Sweden is a country with a population of 10 million, where all residents have a unique personal identification number which allows for cross-linking between health care registers [10]. Health care in Sweden is universally tax-funded and prenatal and maternal care is free of charge for all resident women. All incident cases of cancer in Sweden are registered in the Swedish Cancer Register. The Swedish Cancer Register started in 1958 and since 1984 there is a double reporting routine, where both clinicians and pathologists are obliged by law to report new cases of cancer, ensuring a high degree of completeness and good quality of data [11]. However, there may be a degree of underreporting primarily for indolent cancers [12]. The Inpatient Register covers all discharge diagnoses from hospitalization admissions and was founded regionally in 1964, with complete national coverage from 1987 [13]. The nationwide Outpatient Register was introduced in 2001 and captures the diagnoses of all outpatient visits to specialty clinics. The Medical Birth Register (MBR) was founded in 1973, and includes all pregnancies from gestational week 22 (prior to 2008 week 28). The MBR has a high degree of completeness, 97–99% of all deliveries in Sweden, and captures maternal characteristics as well as antenatal, obstetric, and neonatal data [14]. The Swedish Cancer Register, In- and Outpatient Registers, and MBR are all held by the National Board of Health and Welfare. The Total Population Register, held by Statistics Sweden, includes all individuals residing in Sweden [15]. The Swedish Multi-Generation Register, is based on the Total Population Register, and includes all individuals born after 1932, and who have resided in Sweden at any time-point after 1961, with links to parents residing in Sweden.

### Study participants

The source of the study population is the Multi-Generation Register, thus including women in Sweden born after 1932. This was linked to MBR to identify pregnancies in these women. Due to the properties of MBR, only pregnancies having reached week 22, before 2008 week 28, were included, according to the prevailing distinction between miscarriage and childbirth. From this study base of pregnancies, all pregnancies occurring 1973–2018 where the mother had a diagnosis of MPN were included in the study. One control pregnancy was randomly selected from the same study base for each pregnancy in an MPN patient, matched by maternal age, calendar year of pregnancy, number of previous deliveries, and whether it was a singleton or duplex pregnancy. For women with MPN who had more than one pregnancy, we matched a separate non-MPN control pregnancy to each pregnancy. Other hematologic malignancies in the mother prior to the index pregnancy were an exclusion criterion for pregnancies both in MPN patients and controls.

A diagnosis of MPN was defined as having a diagnosis of PV, ET, PMF, or MPN-U obtained at age 16 or older, in the Swedish Cancer Register (from 1958), the Inpatient Register (from 1964) or in the Outpatient Register (from 2001). In women where the MPN diagnosis was retrieved from Outpatient Register, two separate occasions with an MPN diagnosis were required. Since MPN is a slowly occurring disease that may go undiagnosed for a certain time before the diagnosis is established, we included pregnancies where the mother was diagnosed before pregnancy, during pregnancy or in the immediate post-partum period (60 days). Incidence of pregnancies in women with MPN per 100 000 pregnancies was calculated 2007–2017, however, in the incidence estimation we only included women where a diagnosis of MPN was established prior to pregnancy.

Ethical permission was granted from the Ethical Review Board, informed consent was waived since there was no contact with the study participants.

### Pregnancy, maternal, and child outcomes

Data on gestational length, birthweight, and delivery was collected from the MBR. Preterm birth was defined as birth prior to gestational week 37 + 0, moderate preterm gestational week 32 + 0 to 36 + 6, very preterm gestational week 28 + 0 to 31 + 6, and extremely preterm as born prior to gestational week 28 + 0. The best method of dating the pregnancy was used, ultrasound dating when available, otherwise last menstrual cycle data. Iatrogenic preterm birth was defined as prelabor cesarean section or induced labor prior to week 37 + 0. Low birthweight was defined is birthweight <2500 g, and very low birthweight as <1500 g. Small for gestational age in the MBR is defined according to Marsal et al., as −2 standard deviations in birthweight in relation to gestational length, and is only applicable in singleton pregnancies [16]. Stillbirth was defined as fetal loss from week 22, before 2008 from week 28. Vaginal delivery included both spontaneous and operative vaginal deliveries. Fetal malformations and chromosomal abnormalities were defined as any diagnosis Q00-99 in the child according to International Classification of Diseases (ICD) version 10, or corresponding codes in earlier ICD versions, registered in the MBR.

Previous spontaneous abortions and duration of infertility prior to the index pregnancy were self-reported data also retrieved from MBR. Information on preeclampsia, eclampsia, and HELLP (hemolysis, elevated liver enzymes, and low platelet count) was obtained using ICD codes from MBR Information on other maternal outcomes including transfusion, thrombosis, and bleeding was obtained using ICD codes from the Inpatient Register, Outpatient Register, and MBR, and were captured from start of pregnancy to end of immediate postpartum period (8 weeks post-delivery). Pregnancy-related bleeding was retrieved from MBR, and non-pregnancy-related bleeding was retrieved from Inpatient and Outpatient Register. The variables recorded in the MBR have changed over time, and some information is not available for all years, also earlier ICD-versions are in some variables not specific enough; data with historical truncations include transfusions (available from 1998), severe postpartum hemorrhage >1000 ml (1998), previous infertility and spontaneous abortions (1982), onset of labor (induction, spontaneous start, prelabor cesarean section; 1990), BMI (1982). Percentages regarding these variables indicate proportion of individuals from that year and onwards.

### Statistical methods

The entire cohort of MPN pregnancies and their corresponding controls were included in the descriptive analysis. For the comparative statistical analysis, we restricted to first pregnancy after MPN diagnosis to avoid a mix of dependent and independent data. *P* values for categorical data were calculated using two-sided Fisher exact test, *p* < 0.05 was considered significant. The matched cohort design ensured that the cohort was balanced and that there was no confounding in the exposure-outcome associations (MPN status and pregnancy outcomes), hence the matching was ignored in the analysis [17]. Missing data were excluded in analysis of numerical data, and in categorical data, missing was considered absence of an event. As a sensitivity analysis, outcomes are reported separately for the different registered sources of MPN diagnosis.

All statistical analyses were performed in Stata, version 16 (Stata Corp, Texas, United States), and SAS software, version 9.4 (SAS Institute Inc. Cary, NC United States).

## Results

### Study population characteristics

**Fig. 1:**
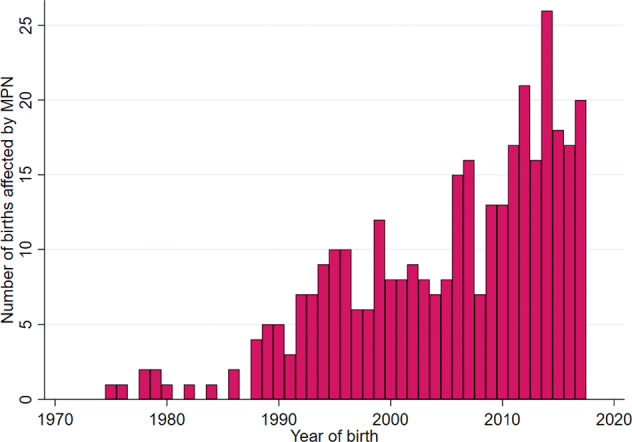
Childbirth in MPN patients per calendar year. Number of births in women with MPN per calendar year in Swedish registers 1973–2018. MPN = myeloproliferative neoplasm.

We identified 342 pregnancies in 229 women with MPN, of which 337 were singleton and five duplex pregnancies, with equal numbers in controls. Forty-three pregnancies occurred in patients with PV, 238 in ET, 33 in PMF, and 28 in MPN-U (Table 1, Supplementary Table 1). Median maternal age at pregnancy was 32 years, interquartile range (IQR) 29–35 years. Median body mass index (BMI) was similar between MPN mothers and control mothers at 24 kg/m^2^. Median time between MPN diagnosis and delivery was 3.7 years, IQR 1.3–7.7 years. Median calendar year of child birth was 2007 (Fig. 1). Eighty-nine pregnancies (26%) were identified in women included through the Swedish Cancer Register, 159 (46%) from the Inpatient Register, and 94 (27%) from the Outpatient Register (Supplementary Table 2). In the MPN cohort of all pregnancies, 26% reported at least one previous miscarriage, and 4% reported at least three previous miscarriages prior to index pregnancy, corresponding percentages for controls were 20% and 2%, respectively (from 1982, *n* = 335). When restricted to first pregnancy after MPN diagnosis, the difference in proportions with a history of at least one or three or more miscarriages was not significant. Twelve percent of all MPN pregnancies were preceded by at least one year of self-reported involuntary childlessness, compared to 10% in control pregnancies.

**Table 1:** Descriptive statistics of study population.

	*All identified pregnancies*		*First pregnancy after MPN diagnosis*	
	MPN patients *n* = 342 (%)	Controls *n* = 342 (%)	MPN patients *n* = 229 (%)	Controls *n* = 229 (%)
Singleton	337 (99)	337 (99)	225 (98)	225 (98)
Duplex	5 (1)	5 (1)	4 (2)	4 (2)
Polycytemia vera	43 (13)	-	27 (12)	-
Essential thrombocythemia	238 (70)	-	158 (69)	-
Myelofibrosis	33 (10)	-	24 (10)	-
MPN-Unclassifiable	28 (8)	-	20 (9)	-
MPN diagnosis prior to pregnancy	273 (80)	-	160 (70)	-
MPN diagnosis during pregnancy or postpartum period	69 (20)	-	69 (30)	-

Pregnancies in patients with MPN and their matched control pregnancies (matched on maternal age, singleton or duplex, year of pregnancy, previous number of pregnancies).

*MPN* myeloproliferative neoplasm.

In 273 (80%) of the MPN pregnancies, the MPN diagnosis was established prior to pregnancy, in 69 (20%) the diagnosis was first recorded during pregnancy or within 60 days postpartum. During 2007–2017, there were in total 1 230 380 deliveries in Sweden, yielding an incidence of MPN delivery of 12.2 per 100,000 deliveries, including only pregnancies in patients where the MPN diagnosis was established prior to the start of pregnancy.

### Neonatal outcomes: gestational age, birthweight, morbidity, stillbirth, and mortality

Preterm birth was more common in women with MPN compared to controls (Table 2). This was consistent in all degrees of preterm birth, with higher numbers of moderate preterm, very preterm, and all cases of extremely preterm being in pregnancies in women with MPN compared to control pregnancies. When restricting to first pregnancy after MPN diagnosis (*n* = 229) the difference was significant, 14% in women with MPN and 4% in controls, *p* < 0.001, as was moderate preterm. Of the 37 preterm deliveries in MPN patients from 1990 and onwards, 15 were registered as prelabor cesarean section, and 4 as inductions, summing up to 19 (51%) iatrogenic preterm birth. There were 14 preterm deliveries during the same years in controls, of which 2 (14%) were iatrogenic preterm births (both prelabor cesarean sections). The increase of iatrogenic preterm birth in MPN compared to controls was significant (*p* = 0.001). Maternal diagnoses listed in MBR for the MPN patients experiencing iatrogenic preterm birth include intrauterine growth restriction (*n* = 5), severe preeclampsia (*n* = 3), moderate preeclampsia (*n* = 2), placental abruption (*n* = 2), amongst other diagnoses.

**Table 2:** Outcomes of the newborn child: gestational age, birthweight, and mortality.

	*All identified pregnancies*		*First pregnancy after MPN diagnosis*		
	MPN patients *n* = 342 (%)	Controls *n* = 342 (%)	MPN patients *n* = 229 (%)	Controls *n* = 229 (%)	*p* value
Low birthweight <2500 g	29 (8)	11 (3)	22 (10)	10 (4)	**0.042**
Very low birthweight <1500 g	7 (2)	0 (0)	7 (3)	0 (0)	**0.015**
Small for gestational age	15 (4)	7 (2)	11 (5)	7 (3)	0.472
Preterm <week 37	42 (12)	14 (4)	33 (14)	10 (4)	**<0.001**
Moderate preterm, week 32–37	31 (9)	12 (4)	22 (10)	9 (4)	**0.024**
Very preterm, week 28–31	6 (2)	2 (0.6)	6 (3)	1 (0.4)	0.122
Extremely preterm, <week 28	5 (1)	0 (0)	5 (2)	0 (0)	0.061
Iatrogen preterm^a^	19 (6)	2 (0.6)	16 (8)	2 (1)	**0.001**
Spontaneous preterm^a^	18 (6)	12 (4)	13 (6)	8 (4)	0.371
Low birthweight, term	6 (2)	4 (1)	3 (2)	4 (2)	1.000
Fetal malformations and chromosomal abnormalities	15 (4)	10 (3)	9 (4)	7 (3)	0.800
Stillbirth	2 (0.6)	0 (0)	1 (0.4)	0 (0)	1.000
Neonatal death	1 (0.3)	1 (0.3)	1 (0.4)	1 (0.4)	1.000

*P* value is calculated only in first pregnancy after MPN diagnosis, Fisher exact test was used and *p* < 0.05 considered significant (bold). Neonatal death is defined as a live birth, with death occurring between day 0 and 28.

*MPN* myeloproliferative neoplasm.

^a^Variables where data are only available from 1990 and onwards, total number of patients and controls from 1990 are 322, when restricted to first pregnancy 212.

Women with MPN experiencing preterm birth were of similar median age, BMI, and previous numbers of miscarriages as women with MPN and term births. Three preterm births were in patients with PV, 29 in ET, 4 in patients with PMF, and 6 in patients with MPN-U (Supplementary Table 1). The proportion of pregnancies in MPN patients with preterm birth was similar in patients diagnosed with MPN prior to pregnancy (12%) as those diagnosed during pregnancy or postpartum period (13%). There was a tendency towards a higher proportion of preterm birth in MPN patients during the earlier part of the study period. In the period 1973–2006, 16% (*n* = 25) of pregnancies ended preterm, compared to 9% (*n* = 17) during 2007–2018. When restricting to first pregnancy after MPN diagnosis, this tendency was not statistically significant (*p* = 0.093). In control pregnancies, the proportion of preterm birth was similar at 4–5% in both calendar periods.

**Fig. 2:**
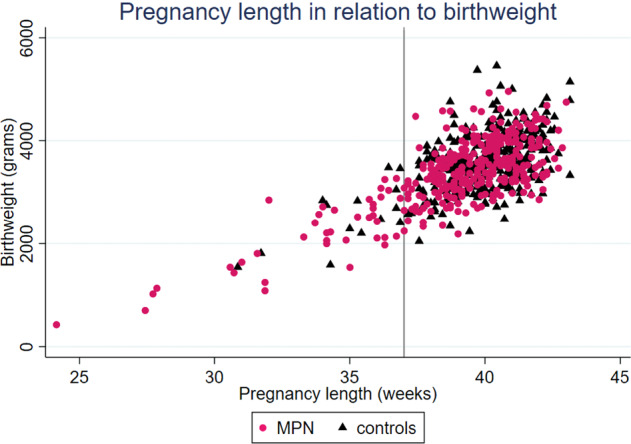
Birthweight in relation to pregnancy length in all 342 pregnancies. Pregnancy length in weeks, birthweight in grams. The vertical line marks beginning of week 37, from which pregnancy is considered term. MPN = myeloproliferative neoplasm.

In keeping with the higher incidence of preterm birth, low birthweight (<2500 g) was also more prevalent in pregnancies in MPN patients (Table 2). There was no significant difference in newborns born small for gestational age. Median weight of newborns in all 342 pregnancies was 3 428 g in MPN mothers compared to 3573 g in controls. When excluding preterm births, the median birthweight was 3505 g vs 3595 g. Birthweight in relation to gestational length at delivery is shown in Fig. 2.

Apgar score [18] at 5 min was similar between neonates to MPN mothers and controls, with 80% of neonates to MPN mothers and 82% of controls having a 5 min Apgar score of 10. There were few events of stillbirth, two (0.6%) in women with MPN and none in a control pregnancies (non significant difference). Two newborns died on their second day of life, one born to a mother with MPN and one to a control. Malformations occurred in 23 neonates, 13 born by mothers with MPN and 10 born by controls, while chromosomal abnormalities occurred in 2 pregnancies in mothers with MPN and none among controls. When restricting to first pregnancy, proportion born with malformations and chromosomal abnormalities was not significantly different.

### Maternal complications: thrombosis, bleeding, and preeclampsia

Venous thrombosis was an overall rare event, with only three events (1%) in MPN patients compared to none in controls (Table 3). When restricted to first pregnancy this was not significant. There were no reported cases of arterial thrombosis in MPN patients or in controls. Pregnancy-related bleeding during the puerperium was recorded in MBR in 47 (14%) women with MPN and 27 (8%) without MPN. Intrapartum or postpartum bleeding >1000 ml was insignificantly increased in MPN 24 (9%) vs 14 (5%) in controls (*p* = 0.423) as were required transfusions 20 (8%) vs 9 (3%) (*p* = 0.212). During pregnancy, not associated with delivery, there were four bleeding events related to pregnancy recorded in the patient registers for the MPN women and one in controls. Regarding non-pregnancy-related bleedings, in women with MPN there was one event of hematemesis registered during pregnancy, and two events of unspecified bleeding during the postpartum period, no non-pregnancy-related bleeding was registered in controls.

**Table 3:** Maternal complications: thrombosis, bleeding, and preeclampsia.

	*All identified pregnancies*		*First pregnancy after MPN diagnosis*		
	MPN patients *n* = 342 (%)	Controls *n* = 342 (%)	MPN patients *n* = 229 (%)	Controls *n* = 229 (%)	*p* value
Pregnancy-related bleeding	47 (14)	27 (8)	33 (14)	21 (9)	0.110
Bleeding during delivery or postpartum >1000 ml^a^	24 (9)	14 (5)	16 (9)	11 (7)	0.423
Transfusion^a^	20 (8)	9 (3)	16 (10)	9 (5)	0.212
Any thrombosis	3 (1)	0 (0)	2 (1)	0 (0)	0.499
Thrombosis, arterial	0 (0)	0 (0)	0 (0)	0 (0)	1.000
Preeclampsia, HELLP, gestational hypertension	19 (6)	13 (4)	16 (7)	11 (5)	0.428

*P* value is calculated only in the first pregnancy after MPN diagnosis, Fisher exact test was used and *p* < 0.05 considered significant. MPN = myeloproliferative neoplasm. HELLP = hemolysis, elevated liver enzymes and low platelet count.

^a^Variables where data are only available from 1998 and onwards, total number of patients and controls from 1998 is 265, when restricted to first pregnancy 169.

The proportion of pregnancies complicated by preeclampsia, HELLP syndrome, or gestational hypertension did not differ between MPN and control mothers.

### Delivery

Delivery by cesarean section was more common in MPN patients than controls, 31% compared with 16% which was statistically significant when restricted to first pregnancies (*p* < 0.001) (Table 4). Induction (20% vs 13%) and prelabor cesarean section (18% vs 9%) were significantly more common in pregnancies in MPN patients than in controls. After spontaneous labor onset (from 1990), 9% were delivered by cesarean section in women with MPN and in 5% in controls (not significant). In vaginal deliveries (from 2006) 30% of MPN patients received epidural anesthesia compared to 33% of controls.

**Table 4:** Mode of labor onset and delivery.

	*All identified pregnancies*		*First pregnancy after MPN diagnosis*		
	MPN patients *n* = 342 (%)	Controls *n* = 342 (%)	MPN patients *n* = 229 (%)	Controls *n* = 229 (%)	*p* value
Onset of labor					
Spontaneous onset^a^	192 (60)	245 (76)	125 (59)	164 (77)	**<0.001**
Induction^a^	64 (20)	41 (13)	44 (21)	24 (11)	**0.012**
Cesarean section before labor onset^a^	58 (18)	29 (9)	35 (17)	20 (9)	**0.042**
Missing data, mode of onset^a^	8	7	8	4	**-**
Mode of delivery					
Delivery by cesarean section	106 (31)	54 (16)	73 (32)	40 (17)	**<0.001**
Vaginal delivery	236 (69)	288 (84)	156 (68)	189 (83)	**-**

*P* value is calculated only in first pregnancy after MPN diagnosis, Fisher exact test was used and *p* < 0.05 considered significant (bold). Vaginal delivery includes all deliveries where cesarean section was not indicated.

*MPN* myeloproliferative neoplasm.

^a^Marks variabels where data are only available from 1990 and onwards, total number patients is then 322 patients and controls, when restricted to first pregnancy 212 patients and controls.

## Discussion

In this population-based study of pregnancies in MPN patients, we found that preterm birth, iatrogenic preterm birth, and low birthweight were significantly more common in pregnancies in MPN patients than in controls. Newborns to MPN mothers had a lower birthweight than those of controls, however, the majority of low birthweight and very low birthweight neonates were likely attributable to preterm birth. Other markers of newborn health were similar, such as Apgar score at 5 min and neonatal mortality. Stillbirth was rare.

Our study suggests a higher incidence of MPN pregnancy of 12.2 per 100,0000 deliveries, compared to 3.2 reported by Alimam et al. Population-based studies are rare, hence there are few other estimations of incidence to use for comparison [7]. An important reason for the increased number of pregnancies in women with MPN over time in our data depicted in Fig. 1 is the addition of Outpatient (2001) and Inpatient Registers (1987). The increasing trend may also be due to an increased incidence of MPN, where facilitated early MPN diagnosis with molecular markers and revised diagnostic criteria may be contributory [2, 19]. There may also be a true increasing trend of pregnancies in women with MPN over time due to an ongoing trend of higher maternal ages [20–22]. Our study, with a population-based approach, suggests that pregnancy in women with MPN may be more common than previously reported.

Preterm birth has a large impact on neonatal and pediatric morbidity and mortality. In the general population, 30–35% of preterm births are indicated for maternal or fetal reasons, and these babies are delivered by cesarean section or induction of labor, while 65–70% are spontaneous preterm births. It has been suggested that spontaneous preterm birth is caused by inflammatory changes; infectious or immunologic, ischemia, overdistension of the uterus, stress, or hemostatic factors [23]. The pathophysiology of the increased proportion of preterm birth in MPN remains to be elucidated, all these pathways are plausible, in particular inflammatory or hemostatic factors. Our data suggest a significant increase of iatrogenic preterm birth in MPN patients compared to controls, but not a significant increase of spontaneous preterm birth. There is not sufficient information in the registers to determine whether iatrogenic preterm birth is explained solely by an increased risk of obstetric complications or if the awareness of the MPN or increased monitoring of the pregnancy due to MPN may have interfered with the decision to end the pregnancy preterm. The risk of preterm birth in women with MPN has previously been mentioned in several smaller studies, as has intrauterine growth restriction. Alimam et al. reported that 85% of births in MPN pregnancies were at term, which comply well with our results [7]. Other studies primarily on pregnancy in patients with ET have shown various results, where Rumi et al. reported 13% preterm birth, How et al. 7% and Gangat et al. 2% [4, 24, 25]. In the general Swedish population, the rate of preterm (5%) and very or extremely preterm (1%) births have been stable over time (1985–2013) [26].

The proportion of MPN patients experiencing thromboembolism during pregnancy or postpartum period (1%) is lower to than what was reported by Skeith et al. in patients with ET [8]. There was a trend towards pregnancy-related bleeding occurring more frequently in pregnant women with MPN than controls, but this difference was not significant, neither was there any significant difference in severe postpartum hemorrhage (>1000 ml) or transfusion requirement during postnatal care. A slight increase in bleedings would be expected considering both the generally increased risk of bleeding in MPN and treatment recommendations during pregnancy. There were significant differences in both mode of labor onset with more induction and prelabor cesarean sections, and mode of delivery, with an overall higher proportion of deliveries ended by section in MPN patients compared to controls.

Clinical guidelines for management of pregnancies in MPN are largely based on expert opinion while robust evidence is lacking. The current Swedish guidelines recommend aspirin from first knowledge of pregnancy to birth, then low molecular weight heparin (LMWH) for the postpartum period in all MPN patients. In high-risk MPN pregnancies, defined by history of thrombohemorrhagic events, history of pregnancy loss or other pregnancy complications, cytoreduction with pegylated interferon α and LMWH throughout pregnancy are added [27], similar to management described by Robinson et al., Griesshammer et al. and Gangat et al. [3, 6, 28], and supported by retrospective data [4, 29]. Historically, aspirin was shown to be beneficial in pregnant women with MPN in a small case series in the 1990s [30], and the CLASP study published in 1994 showed that aspirin was well tolerated in pregnant women for prevention of preeclampsia [31], but with a slight increase of postpartum bleeding [32, 33]. Aspirin use in all PV patients became widely accepted after the ECLAP study published in 2004 [34]. The Nordic MPN guidelines first included management of pregnancies in 2009 recommending aspirin throughout pregnancy and LMWH after delivery.

The main strengths of this study were the use of high-quality nationwide registers with prospectively collected data, and the population-based design which minimizes selection bias. Moreover, the study included a large number of pregnancies and all MPN subtypes. Another important strength was the use of matched controls for comparison, which to our knowledge has not been done previously and gives a more accurate comparison of excess risks. Limitations of this study included lack of detailed clinical information in the registers; comorbidities, mutational status, blood counts, and treatment data. There were also uncertainties in reporting MPN cases to the patient registers. Malignant diagnoses are considered more certain when identified through Swedish Cancer Register than in the Patient registers, due to the double reporting routine. To overcome this uncertainty, we required two separate time points for diagnoses when included from the outpatient register, and report main outcomes separately by source of MPN diagnosis (Supplementary Table 2). Another important factor to bear in mind with our study design is that we have included pregnancies from gestational week 22 (before 2008 from week 28). These results are thus applicable to pregnancies in MPN patients having reached that timepoint. Earlier pregnancy loss, prior to weeks 22 and 28, is not captured in these registers and with this study design. As miscarriages were not captured, live birth rate cannot be calculated. Since diagnostic criteria, including distinction of prefibrotic myelofibrosis were modified during the study period, trends over time in relation to subtype should be interpreted with caution.

In conclusion, in this nationwide population-based study of pregnancies in women with MPN, we found that preterm birth, in particular iatrogenic preterm birth, was an important adverse event in women with MPN while thrombotic events were rare. The underlying biology and pathophysiology of how the MPN affects the pregnant woman, the uterus, placenta, and fetus need to be further elucidated. Although our study indicates a higher incidence of childbirth in MPN than previously reported, future systematical studies of optimal treatment strategies during pregnancy in MPN will require international collaboration. In a clinical context, women with MPN need to be monitored carefully during pregnancy. Importantly, the maternal and fetal complication rates were lower than previously reported and the majority of pregnancies in women with MPN that reached week 22/28 ended with a live term birth.

## Supplementary information

Supplementary Table 1. Pregnancy outcomes per MPN subtype in all 342 pregnancies.

Supplementary Table 2. Sensitivity analysis, main outcomes in patients shown separately for each source of MPN diagnosis.

## Data Availability

According to the ethical permission, the data used in this study may not be shared by the authors with a third party. It is accessible by application to the Swedish National Board of Health and Welfare and Statistics Sweden.
