# Supplementary material for: Pregnancy and childbirth outcomes in women with myeloproliferative neoplasms—a nationwide population-based study of 342 pregnancies in Sweden
Source: Leukemia. 2022 Sep 7;36(10):2461–7. doi: 10.1038/s41375-022-01688-w (PMC9522578; doi:10.1038/s41375-022-01688-w)
Supplement: Supplementary file 1 — Supplementary Table 1. Pregnancy outcomes per MPN subtype in all 342 pregnancies. [file 41375_2022_1688_MOESM1_ESM.docx]

**Supplementary Table 1. Pregnancy outcomes per MPN subtype in all 342 pregnancies.** MPN = Myeloproliferative neoplasm, PV = polycythemia vera, ET = essential thrombocythemia, PMF = primary myelofibrosis, MPN-U = MPN Unclassifiable, HELLP = Hemolysis, elevated liver enzymes, low platelets

|  | PV  Number of pregnancies (%) | | ET  Number of pregnancies (%) | | PMF  Number of pregnancies (%) | | MPN-U  Number of pregnancies (%) | |
| --- | --- | --- | --- | --- | --- | --- | --- | --- |
|  | MPN | controls | MPN | controls | MPN | controls | MPN | controls |
| Total | 43 | 43 | 238 | 238 | 33 | 33 | 28 | 28 |
| Low birthweight | 3 (7) | 0 (0) | 19 (8) | 8 (3) | 3 (9) | 1(3) | 4 (14) | 2 (7) |
| Very low birthweight | 0 (0) | 0 (0) | 4 (2) | 0 (0) | 2 (6) | 0 (0) | 1 (4) | 0 (0) |
| Preterm | 3 (7) | 1 (2) | 29 (12) | 10 (4) | 4 (12) | 1 (3) | 6 (21) | 2 (7) |
| Moderate preterm | 3 (7) | 1(2) | 22 (9) | 9 (4) | 2 (6) | 1 (3) | 4 (14) | 1 (4) |
| Very preterm | 0 (0) | 0 (0) | 3 (1) | 1 (0.4) | 1 (3) | 0 (0) | 2 (7) | 1 (4) |
| Extremely preterm | 0 (0) | 0 (0) | 4 (2) | 0 (0) | 1 (3) | 0 (0) | 0 (0) | 0 (0) |
| Stillbirth | 1 (2) | 0 (0) | 1 (0.4) | 0 (0) | 0 (0) | 0 (0) | 0 (0) | 0 (0) |
| Pregnancy-related bleeding | 12 (28) | 1 (2) | 26 (11) | 24 (10) | 6 (18) | 1 (3) | 3 (11) | 1 (4) |
| Any thrombosis | 0 (0) | 0 (0) | 3 (1) | 0 (0) | 0 (0) | 0 (0) | 0 (0) | 0 (0) |
| Preeclampsia, HELLP, gestational hypertension | 2 (5) | 4 (9) | 14 (6) | 6 (3) | 1 (3) | 2 (6) | 2 (7) | 1 (4) |
| Induction (1990) | 12 (34) | 7 (20) | 40 (17) | 26 (11) | 7 (24) | 5 (17) | 5 (18) | 3 (11) |
| Cesarean section | 13 (30) | 0 (0) | 68 (29) | 42 (18) | 14 (42) | 4 (12) | 11 (39) | 8 (29) |
| Median birthweight, term newborns (g) | 3448 | 3433 | 3555 | 3600 | 3470 | 3740 | 3360 | 3600 |
