# Supplementary material for: Pregnancy and childbirth outcomes in women with myeloproliferative neoplasms—a nationwide population-based study of 342 pregnancies in Sweden
Source: Leukemia. 2022 Sep 7;36(10):2461–7. doi: 10.1038/s41375-022-01688-w (PMC9522578; doi:10.1038/s41375-022-01688-w)
Supplement: Supplementary file 2 — Supplementary Table 2. Sensitivity analysis, main outcomes in patients shown separately for each source of MPN diagnosis. [file 41375_2022_1688_MOESM2_ESM.docx]

**Supplementary Table 2. Sensitivity analysis, main outcomes in patients shown separately for each source of MPN diagnosis**. MPN = Myeloproliferative neoplasm, PV = polycythemia vera, ET = essential thrombocythemia, PMF = primary myelofibrosis, MPN-U = MPN Unclassifiable, HELLP = Hemolysis, elevated liver enzymes, low platelets

|  | Cancer Register  Number of pregnancies (%) | | Inpatient Register  Number of pregnancies (%) | | Outpatient Register  Number of pregnancies (%) | |
| --- | --- | --- | --- | --- | --- | --- |
|  | MPN | controls | MPN | controls | MPN | controls |
| Total | 89 | 89 | 159 | 159 | 94 | 94 |
| PV | 18 (20) |  | 15 (9) |  | 10 (11) |  |
| ET | 59 (66) |  | 116 (73) |  | 63 (67) |  |
| PMF | 6 (7) |  | 27 (17) |  | 0 (0) |  |
| MPN-U | 6 (7) |  | 1 (0.6) |  | 21 (22) |  |
| Low birthweight | 10 (11) | 2 (2) | 14 (9) | 5 (3) | 5 (5) | 4 (4) |
| Very low birthweight | 3 (3) | 0 (0) | 4 (3) | 0 (0) | 0 (0) | 0 (0) |
| Preterm | 12 (13) | 3 (3) | 19 (12) | 7 (4) | 11 (12) | 4 (4) |
| Moderate preterm | 8 (9) | 3 (3) | 15 (9) | 6 (4) | 8 (9) | 3 (3) |
| Very preterm | 1 (1) | 0 (0) | 2 (1) | 1 (0.6) | 3 (3) | 1 (1) |
| Extremely preterm | 3 (3) | 0 (0) | 2 (1) | 0 (0) | 0 (0) | 0 (0) |
| Stillbirth | 1 (1) | 0 (0) | 1 (0.6) | 0 (0) | 0 (0) | 0(0) |
| Pregnancy-related bleeding | 13 (15) | 6 (7) | 26 (16) | 11 (7) | 8 (9) | 10 (11) |
| Any thrombosis | 0 (0) | 0 (0) | 3 (2) | 0 (0) | 0 (0) | 0 (0) |
| Preeclampsia, HELLP, gestational hypertension | 4 (5) | 5 (6) | 8 (5) | 5 (3) | 7 (7) | 3 (3) |
| Induction (1990) | 18 (22) | 14 (17) | 27 (18) | 16 (11) | 19 (20) | 11 (12) |
| Cesarean section | 29 (33) | 14 (16) | 45 (28) | 20 (13) | 32 (34) | 20 (21) |
| Median birthweight, term newborns (g) | 3527 | 3562 | 3517 | 3600 | 3490 | 3600 |
